# What Is the Role of Explainability in Medical Artificial Intelligence? A Case-Based Approach

**DOI:** 10.3390/bioengineering12040375

**Published:** 2025-04-02

**Authors:** Elisabeth Hildt

**Affiliations:** Center for the Study of Ethics in the Professions, Illinois Institute of Technology, Chicago, IL 60616, USA; ehildt@iit.edu

**Keywords:** artificial intelligence (AI), machine learning, explainable AI, clinical decision support system (CDSS), ethics, informed consent, medical decision making, autonomy, doctor–patient relationship, automation bias

## Abstract

This article reflects on explainability in the context of medical artificial intelligence (AI) applications, focusing on AI-based clinical decision support systems (CDSS). After introducing the concept of explainability in AI and providing a short overview of AI-based clinical decision support systems (CDSSs) and the role of explainability in CDSSs, four use cases of AI-based CDSSs will be presented. The examples were chosen to highlight different types of AI-based CDSSs as well as different types of explanations: a machine language (ML) tool that lacks explainability; an approach with post hoc explanations; a hybrid model that provides medical knowledge-based explanations; and a causal model that involves complex moral concepts. Then, the role, relevance, and implications of explainability in the context of the use cases will be discussed, focusing on seven explainability-related aspects and themes. These are: (1) The addressees of explainability in medical AI; (2) the relevance of explainability for medical decision making; (3) the type of explanation provided; (4) the (often-cited) conflict between explainability and accuracy; (5) epistemic authority and automation bias; (6) Individual preferences and values; (7) patient autonomy and doctor–patient relationships. The case-based discussion reveals that the role and relevance of explainability in AI-based CDSSs varies considerably depending on the tool and use context. While it is plausible to assume that explainability in medical AI has positive implications, empirical data on explainability and explainability-related implications is scarce. Use-case-based studies are needed to investigate not only the technical aspects of explainability but also the perspectives of clinicians and patients on the relevance of explainability and its implications.

## 1. Introduction: Explainability in Medical Artificial Intelligence

### 1.1. Medical Artificial Intelligence

Recent years have seen an enormous impact of artificial intelligence (AI) on medicine and healthcare, with applications in a broad spectrum of fields such as radiology, dermatology, cardiology, pulmonology, ophthalmology, gastroenterology, and mental health [1,2,3,4,5,6]. AI technologies are expected to fundamentally transform medicine and healthcare in that they have the potential to improve diagnosis and treatment, quantify patient health risks, increase population health, enhance patient experience, and reduce healthcare costs. Particularly in fields like precision medicine and personalized healthcare, the enormous opportunities that AI offers have been stressed. Terms like “augmented intelligence” [3,4] or “augmented medicine” [2] have been used to describe the support AI tools can provide to medical professionals and patients.

AI-driven clinical decision support systems (CDSSs) that support clinicians in managing or delivering patient care are of particular relevance in this context [2,6,7,8,9,10,11]. Examples of CDSS applications include the diagnosis of breast cancer or melanoma, the optimization of drug dosage, or the prediction of response to chemotherapy.

While medical AI applications certainly have enormous potential to improve medicine and healthcare, they also come with considerable risks, downsides, and negative implications. In view of the emerging transformation of the field, a broad spectrum of practical concerns and ethical issues have been raised [3,7,8,10,12,13,14,15,16,17,18,19,20,21,22]. Of central relevance among these is the clinical validation of AI-based tools and the imperative to avoid harm to patients. Issues to consider include accuracy, safety, and efficiency, but also questions related to privacy, data protection, data ownership, fairness, non-discrimination, transparency, explainability, autonomy, accountability, and liability. Safety and reliability of the AI tools are crucial, which requires the proper functioning of the technology and the absence of algorithmic bias. As algorithmic decision making in healthcare presupposes huge amounts of health-related patient data, issues concerning data security and the collection, sharing, and use of data are of vital importance. This includes concerns that patient data are collected, handled or shared without adequate patient informed consent or that security breaches and cyberattacks may jeopardize patient data and patient safety. Steps need to be taken to secure data privacy which implies that patients have control over their health-related information and to prevent privacy breaches and confidentiality violations.

In addition, the use of AI-based CDSSs raises complex questions related to clinical communication, informed consent, and changes in the roles of medical professionals and doctor–patient relationships, as well as concerns that medical professionals may be replaced by AI technology. Some of these issues are particularly salient in the case of black box models, i.e., highly complex models that are not intelligible to human users. Most of the best performing models, such as support vector machines and neural networks including deep neural networks, are opaque from a technical perspective [9,23]. With black box CDSSs, the functioning of which is incomprehensible to human users, it is difficult for users to know when to rely on the tool output and when to follow their own decision making. The opacity of the tool further exacerbates issues and concerns relating to harm prevention, bias, fairness, autonomy, accountability, liability, and doctor–patient communication, among others.

Jenna Burrell [24] distinguished between various forms of AI-related opacity: opacity from intentional corporate or state secrecy; opacity as technical illiteracy, i.e., opacity for people who lack the specialist knowledge and skills required to understand the functioning of algorithms and tools; and opacity that arises from the characteristics and complexity of machine learning (ML) algorithms [24].

The increasing effort to reduce the third type of opacity, which could be called technological opacity [25], has facilitated the development of explainable AI (xAI) in recent years.

### 1.2. Explainability

Explainability in AI technology can be broadly described as allowing users to understand how the technology arrives at its predictions or recommendations. Explainability can refer to the model as a whole (global explainability) or specific predictions (local explainability) [9,25,26,27,28,29,30].

The concept of explainability in AI is not well defined, however. For example, Julia Amann and colleagues give the following definition [26] (p. 2): “In its essence, explainability can be understood as a characteristic of an AI-driven system allowing a person to reconstruct why a certain AI came up with the presented predictions”. Similarly, Tianhua Chen et al. characterize explainability as “the ability to present and adequately justify the rationale behind decisions or recommendations” [31] (p. 9). Rita Matulionyte and colleagues [25] (p. 158) prefer a more technical understanding of ‘explainability’ and see it as an “explanation of *how* an AI system functions, namely, how it generates outputs, which in most cases will require using specific explainability approaches or techniques”.

From a technical perspective, a distinction can be made between two different forms of explainability methods: ante hoc and post hoc explanation methods [9,23,26,28,29,30,32]: Ante hoc methods are inherently explainable methods (explainability by design; white box approaches; glass-box models). They include linear and logistic regression, decision trees, rule-based learners, fuzzy inference systems, k-nearest neighbors, and Bayesian models. In contrast, post hoc explanation methods provide local explanations for specific decisions. Here, a second model is used to provide approximate explanations of a black box model.

To distinguish between the two types of approaches, the term “interpretable AI” has been used for the first approach, and the term “explanation” for the second approach involving post hoc explanations (see for example [28,32]). The distinction between these two approaches is very important and will be further discussed below in the context of the use cases (see Section 3.3). To avoid terminological confusion, however, this article will use the terms “explainability” and “explainable AI” in a more general way to characterize AI that provides some form of insight, justification, or explanation of how it generates its output.

A number of terms and concepts exist that are similar to the concept of explainability, such as explicability, interpretability, intelligibility, or transparency [9,25,27,33,34], (see also the above distinction between interpretable and explainable algorithms). Overall, the debate is complicated by the fact that none of these concepts are well defined [9,25,28,34].

Notwithstanding this terminological difficulty, explainability (or the related concepts of explicability or interpretability) has been considered a central principle of ethical AI, alongside principles like respect for human autonomy, prevention of harm, and justice [34,35,36,37]. In line with this, explainability (explicability, interpretability, depending on the exact definition) is among the concepts and principles of central relevance in a broad spectrum of AI-related ethical guidelines and codes [38,39,40].

The central relevance of explainability for ethical AI stems from the strong direct connections between explainability and human decision making, agency, and accountability. Human autonomous decision making and agency require adequate knowledge of the available options. In the context of AI applications, explainability can be seen as significantly enhancing knowledge and supporting user autonomy, in that the explanations given by the tool contribute to or provide a basis for well-informed, autonomous human decision making. In contrast, when human users rely on black box AI output, they do not know how the model output was generated. This lack of relevant information forecloses well-informed choice and autonomous decision making and only leaves the option for human users to decide whether to rely on a black box AI system and its output.

### 1.3. On the Relevance of Explainable AI in Medicine and Healthcare

Particularly in medical and healthcare contexts, explainability has been considered an important characteristic of AI technology that can help increase the acceptance of AI tools [9,25,26,28,41]. Against this background, in recent years, a broad spectrum of approaches and AI-based CDSSs that involve explainable AI algorithms have been developed [9,29,42,43,44,45,46,47].

Several strong reasons have been cited for the importance of explainability in medical AI, including AI quality and transparency; bias detection; quality of decision making; autonomy and doctor–patient relationships; trust and trustworthiness; fairness; responsibility, accountability, and liability [9,20,25,26,48]. Several authors have stressed that explainability in medical AI helps to avoid or mitigate potential negative implications of AI-based tools on doctor–patient relationships, autonomy, and informed consent. As compared to black box models, explainability in AI is seen to increase trust and facilitate shared clinical decision making and patient-centered medicine [16,26,41,48,49].

Other authors, however, have questioned the relevance of explainability for medical AI applications [25,48,50,51]. They only see a limited role for explainable AI and doubt whether explainability is needed to improve the quality of CDSSs, increase trust, preserve autonomy, support doctor–patient relationships, or facilitate the adoption of AI tools in clinical contexts.

**Figure 1:**
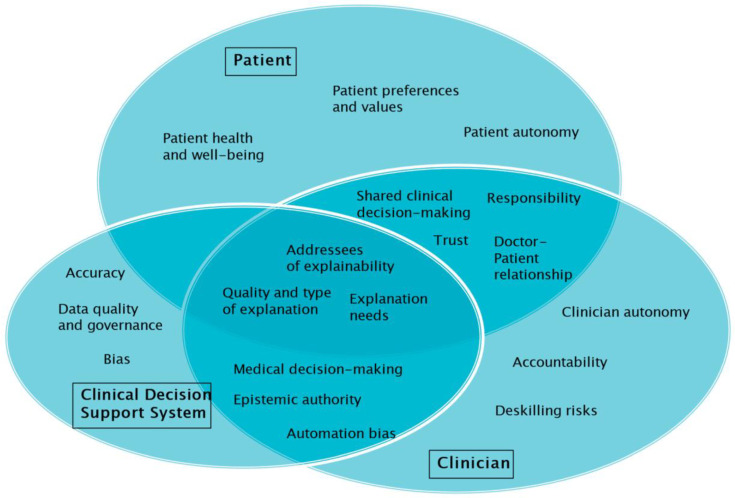
Role of explainability: Interplay between clinical decision support system (CDSS), clinician and patient.

Figure 1 provides an overview on the role of explainability in CDSSs and the interplay between CDSS, clinician, and patient.

### 1.4. Motivation for This Research and Research Questions

As delineated above, the interdisciplinary ethics discussion on the role of explainability in medical AI has been a discussion that focuses on explainability in medical AI in general, most often without taking a detailed look at specific forms of explainability and use contexts.

Against this background, in various ways, the general dispute about the relevance of explainability in AI-driven CDSSs seems to be a currently unresolved controversy between two opposed camps. One reason for this is the lack of empirical data. As Julia Amann and colleagues put it [28] (p. 5): “There is considerable uncertainty about the expected utility and appropriate implementation of explainability in AI-based CDSSs”.

In addition, it is certainly not adequate to take a “one size fits all” approach to explainability in CDSSs. The role of explainability in AI-based CDSSs varies, depending on the tool, its application, and its use context. In different use cases, the type and amount of explanation considered useful may vary considerably. The same may hold for the relevance of explanations.

To better understand the role and relevance of explainability in AI-driven CDSSs, a case-based analysis is needed that allows for a more detailed reflection on the ethical aspects of explainability in the context of specific applications.

This article seeks to fill this gap of context-specific ethics reflection by providing a case-based discussion of four use cases that involve different forms of explainability. In the context of the four use cases, the following research questions will be addressed: Who needs explainability in medical AI and why? What type of explainability is needed? What is the relevance of explainability as compared to accuracy? How do explainable CDSSs influence medical decision making and the roles and responsibilities of medical professionals? What is the role of explainability in medical AI when it comes to considering individual values and preferences? How do explainable CDSSs influence doctor–patient relationships, patient autonomy and informed consent?

After an introduction of the four use cases, these research questions will be addressed in the context of the respective use cases. In this, the focus is on the ethical aspects of explainability in CDSSs.

## 2. Four Use Cases

Four examples of AI-driven CDSSs will be introduced and discussed to highlight explainability’s role in medical AI. The four use cases were chosen to cover a broad spectrum of methods, applications, and use contexts.

The first example is a machine learning model designed as a supportive tool to recognize cardiac arrest in emergency calls. It does not provide any explanation of how it comes up with its output, i.e., it functions as a black box. Here, it is especially the lack of explanation that will be discussed (Section 2.1). The second use case is about a tool designed to support the early diagnosis of Alzheimer’s disease (AD). It involves post hoc explanations that indicate the relevance of the various brain regions for the predictions (Section 2.2). The next example depicts an AI tool developed to support the diagnosis of adult attention deficit hyperactivity disorder (ADHD). The hybrid tool relies in part on a knowledge representation model that provides explanations in line with medical knowledge (Section 2.3). The fourth use case introduces a model developed to simulate moral decision making. It involves a fuzzy cognitive map with the nodes representing relevant medical parameters and ethical concepts (Section 2.4).

### 2.1. Lack of Explanations: Machine Learning-Based Identification of Cardiac Arrests in Emergency Calls

The first example is a machine learning framework designed as a supportive tool to recognize cardiac arrest in emergency calls [52,53,54]. In health-related emergency calls, emergency medical dispatch centers face the challenge of correctly identifying out-of-hospital cardiac arrest (OHCA) cases among a large number of calls. In the case of cardiac arrest, cardiopulmonary resuscitation must be initiated immediately to save lives. However, fast identification of these cases has proven difficult.

To help medical dispatchers identify cases of out-of-hospital cardiac arrest, a ML framework based on a convolutional deep neural network was developed and tested by relying on emergency calls to the Emergency Medical Dispatch Center Copenhagen. The central goals of this approach were to speed up the identification process and increase the identification rate of out-of-hospital cardiac arrests.

The ML model was trained and tested by using unedited recorded calls to the 112 emergency line. No other personal data were used. The audio was transcribed to text by a language model based on a convolutional deep neural network. The language model output was then fed to a classifier, which made a prediction of whether to consider the call a case of cardiac arrest. The tool did not provide any explanations on how it arrived at its predictions. It “listened in” on the communication between the caller and the dispatcher without asking any questions to the caller or dispatcher.

In a retrospective study with recorded calls [52], compared with medical dispatchers, the system was found to have a significantly higher sensitivity with lower, albeit similar, specificity. The tool had a lower positive predictive value than the dispatchers. Also, it could recognize cardiac arrest cases faster than the dispatchers. As a Danish language model was used, there were some limitations with callers who had dialects or accents or did not speak Danish [52,54].

The ML framework was also used in a randomized clinical trial in which it analyzed emergency calls in real time and delivered decision support to medical dispatchers [53]. The model “listened” to the calls and provided an alert to the dispatchers in cases that were classified as cardiac arrest. The dispatchers were free to consider the ML system’s output in the decision-making process, to agree or disagree with it, or to ignore it. In case they suspected a cardiac arrest, the dispatchers provided the caller with instructions for immediate cardiopulmonary resuscitation and sent out an ambulance.

As the results of this clinical trial show, the dispatchers and the ML model missed the same patients, with the ML model missing fewer. However, even though the ML model had higher sensitivity than the dispatchers and was able to identify cardiac arrests faster, there was no significant increase in the number of identified cases of out-of-hospital cardiac arrests when the ML model supported the dispatchers, as compared to the dispatchers alone [53]. This study indicated that being alerted to a suspected cardiac arrest by the ML system did not significantly influence the behavior of the dispatchers who responded to the call. It seems that the dispatchers tended to neglect the alert given by the tool in their decision-making process [53].

While it is important to stress that there is no empirical study that investigated the reasons for this apparent lack of compliance with the decision support tool output, it is plausible to assume that lack of effective communication and training when implementing the tool and lack of trust in the tool are relevant factors [28,53,54]. Accordingly, lack of explainability could have played an essential role in the dispatchers’ hesitancy to rely on the tool. The fact that the tool functions as a black box implies that the dispatchers had no way to determine whether it was right. It gives an alarm but does not provide any explanation of how it comes up with its classification. In their detailed analysis, Amann et al. [28] suggest that adding explainability could be an efficient way to increase compliance and trust. Another potentially relevant factor for the lack of compliance could be the tool’s relatively high number of false alerts, which comes with the risk of dispatchers sending out ambulances to false-positive cases. This may detract urgently needed resources from other patients.

### 2.2. Post Hoc Explanations: AI-Based Prediction of Alzheimer’s Disease

Alzheimer’s disease (AD) is one of the most prevalent progressive neurodegenerative disorders worldwide, resulting in irreversible brain damage [55,56]. The disorder is characterized by memory loss, cognitive impairment, and dementia progressing throughout the course of the disease. Currently, there is no cure available. Existing treatments only alleviate symptoms.

A broad spectrum of approaches exists for detecting AD in its early stages, including brain imaging techniques such as magnetic resonance imaging (MRI) or positron emission tomography (PET) [57,58]. Recently, approaches involving machine learning and deep learning algorithms have been developed for AD prediction [59,60,61,62]. In addition to allowing the early diagnosis of early stages of AD, an important goal of these studies is to increase the accuracy of the AD classification. Among them, a number of studies used explainable AI algorithms like LIME (Local Interpretable Model-Agnostic Explanations) or SHAP (SHapley Additive exPlanations) that provide post hoc explanations for the AD classifications of black box models [9,23,29,63].

For example, Hamza Ahmed Shad and colleagues conducted a study involving explainable AI that sought to increase the classification accuracy and achieve an early AD prediction by using several Convolutional Neural Networks [64]. The team aimed to classify patient data into four classes based on MRI imaging data: non-demented, very mildly demented, mildly demented, and moderately demented. In this, one of their goals was to distinguish between Mild Cognitive Impairment (MCI) and early AD. The study used three different Convolutional Neural Network (CNN) models, Resnet50, VGG16, and Inception v3. Each of these models obtained a categorical accuracy of over 82% with the testing dataset.

To determine which regions are relevant for classification, Shad and colleagues [64] combined this approach with LIME, an algorithm that seeks to explain the predictions of any classifier by approximating it locally with an interpretable model [65]. LIME provides explanations that indicate how much the various features contribute to the predictions. However, as LIME only approximates the other models, the LIME explanations may not accurately represent the inner workings of the original models.

In the study by Shad et al. [64], LIME was used for each of the CNN models. The LIME regional explanations for patient classification varied between the CNN models. While some overlap in the indicated brain areas existed between the models, for each of the CNN models, LIME also indicated additional, different brain areas as relevant for the classification of patients as non-demented, very mildly demented, mildly demented, and moderately demented.

Even though post hoc explanations indicate correlation and not causation (see Section 3.3), a result like this is counterintuitive. From a medical point of view, it would be expected that the explanations provided are in line with medical knowledge. This would imply that for each CNN, the explanations involve the same or at least very similar brain regions as central for AD prediction.

### 2.3. Knowledge-Based Explanations: A Hybrid Model to Support Diagnosis of Adult ADHD

This use case involves a hybrid model developed for AI-supported diagnosis of adult attention deficit hyperactivity disorder (ADHD) [66,67,68]. The tool was developed against the background of increased awareness of adult ADHD, a condition characterized by inattentiveness, hyperactivity, and impulsiveness. Increased awareness of the disorder has come with an increased need for senior psychiatrists to do ADHD assessments. Given a lack of qualified senior specialists, long waiting times of 1–3 years have been seen in the United Kingdom (UK) for adult ADHD diagnosis, leading to underdiagnosis and undertreatment.

To help speed up the process, a group of researchers developed a hybrid AI tool to support the clinical diagnosis of ADHD in adults by automating the diagnosis process [66,67,68]. It supports ADHD diagnosis by separating the patients with complex cases from the clear-cut cases. When used in initial patient consultations, the tool recommends a negative, positive, or inconclusive diagnosis. The consultations of patients with clear negative or positive recommendations can then be concluded by junior clinicians or specialized nurses, with senior specialists assessing the complex cases with inconclusive results. Overall, according to the researchers, by assisting doctors in separating cases based on complexity and getting a broader range of health professionals involved, the AI tool can support faster diagnosis and treatment, reduce waitlists, and allow better allocation of resources [68].

The tool relies on a hybrid approach that integrates a machine learning model and a knowledge model [66,68,69]. The ML model was trained on anonymized data from screening questionnaires and clinical interviews routinely collected from patients. Among the ML algorithms tested in a preliminary research study, a decision tree learning algorithm provided the best results with a predictive clinical performance of approximately 85% [66,69].

While a decision-tree algorithm allows some level of explainability, the main entrance point for explainability in the hybrid tool is the knowledge representation model, which captures the expertise of medical professionals. The knowledge representation model was built based on expert knowledge derived from recommendations by the UK’s National Institute for Health and Care Excellence (NICE) and interviews with an experienced clinician. The knowledge model is based on generic rules that indicate the relevance of the various assessments for ADHD diagnosis. In addition, there are indicators for overlapping conditions such as personality disorder, bipolar disorder, anxiety, depression, or substance abuse. The if-then rules may conflict with each other. For example, in cases when the DIVA (Diagnostic Interview for ADHD in Adults) scores are above threshold, the model’s ADHD diagnosis is “yes”, whereas when they are below threshold, it is “no”, complemented by the rule that if multiple indicators are present, then the decision is inconclusive/‘expert’. Rule prioritization gives the presence of multiple indicators higher priority, so that in case of high DIVA scores and the presence of multiple indicators, the decision is ‘expert’.

The hybrid model combines the ML model and the knowledge model. It is more robust than the individual models alone as both models must align for a yes or no answer. Whenever the two models have different outcomes or the knowledge model outcome is ‘expert’, the hybrid model recommends expert consultation by a senior psychiatrist. The tool does not provide a confidence score. When the hybrid tool was tested at a clinic, its accuracy was determined to be 95% [66] and 93.6% [68].

### 2.4. Explanations Involving Complex Concepts: Simulation of Moral Decision Making in Healthcare

The tool “Algorithmic Advisory System for Moral Decision-Making in the Health-Care Sector” developed by an interdisciplinary group of researchers [70,71] seeks to automate moral decision making. The research team used a fuzzy cognitive map (FCM) that simulates human decision making around complex medical decision-making cases brought before clinical ethics committees. The algorithm output consists of recommendations concerning a medical intervention’s initiation, continuation, or withdrawal. In this, a causal graph represents the decision-making process in which the nodes represent the relevant parameters of the medical case and central ethical concepts. The model was trained using 69 complex medical cases brought before clinical ethics committees and published in the medical ethics literature.

The medical parameters include variables such as the likelihood of the intervention’s positive or negative effects, the patient’s willingness to take risks, the priority of life expectancy versus quality of life, the patient’s age and decision-making capacity, the existence of an applicable advance directive, and similar aspects.

The theoretical concepts involved are beneficence, nonmaleficence, and respect for human autonomy. They were adopted from the principle-based approach developed by Tom L. Beauchamp and James F. Childress [72]. To avoid any health-political and socio-economical background assumptions, justice, the fourth central prima facie principle in the theoretical framework by Beauchamp and Childress, was omitted in the approach. Thus, the model considers only the level of individual patients and ignores any justice or fairness-related aspects. Medical professionals can enter the case-related details and patient preferences themselves or do so together with their patients.

Overall, the FCM comprises 21 nodes and 54 connections. In the network, every node receives weighted input from clearly identified parameters and concepts with which it is causally connected. For example, the autonomy node receives direct input from nodes that represent patient age, patient decision-making capacity, the existence of an advance directive, the availability of the patient’s written preference, or a surrogate’s preference.

The output is a recommendation concerning the intervention in question. Values range between 0 and 1, with 1 meaning strongly in favor of the intervention and 0 meaning strongly opposed to it. In the publication, outputs of 0.5 and above were taken as approval. The tool indicates the relevance of various concepts, such as autonomy, beneficence, nonmaleficence, patient’s best interest, patient’s preference, or surrogate’s preferences, to the recommendation.

The FCM’s nodes and edges have clearly specified human-assigned semantic meanings, and the weights of the causal connections between the nodes are known, making the network highly interpretable. The model is transparent about the factors that causally influence its output. This allows potential users—medical doctors, patients, ethics committees—to see whether the model’s output aligns with their preferences and values.

When the research team evaluated the performance of the algorithm, they found that the predictions deviated from the textbook solutions and the ethicists’ judgement by 0.11 in the training situation and 0.23 in the testing situation. In 92% of the cases in the training and in 75% of the cases in the testing situation, the algorithm agreed with the ethics experts, i.e., made the same recommendation concerning the intervention in question.

The researchers stress that at this stage, they do not aim at providing an operational tool but that their approach is to be seen as proof of concept. They suggest that potential future applications of a similar, more developed tool could include informal guidance on medical ethics dilemmas and educational purposes [70,71].

Automating moral decision making is a highly controversial approach. It comes with a broad spectrum of practical and ethical issues, including the following: Can moral decision making ever be adequately operationalized? Can a tool that is based only on three prima facie principles simulate complex clinical decision making? What are potential implications for doctor–patient relationships and patient autonomy? What is the role of ethics committees and clinical ethicists [73,74,75,76,77]?

## 3. Explainability in the Context of the Use Cases

In what follows, the role of explainability in the context of the use cases will be discussed in more detail. Each of the use cases involves a different type of explainability that comes with a broad spectrum of implications in the respective context. Some of these explainability-related implications are similar in the various use cases, others are use-case specific. All of them need careful consideration.

To reflect on the role and implications of explainability in the context of the use cases, seven explainability-related aspects and themes will be distinguished. These are: (1) The addressees of explainability in medical AI; (2) the relevance of explainability for medical decision making; (3) the types of explanation provided; (4) the (often-cited) conflict between explainability and accuracy; (5) epistemic authority and automation bias; (6) individual preferences and values; and (7) patient autonomy and doctor–patient relationships. Table 1 provides a summary of the results.

### 3.1. Who Needs Explainability in Medical AI?

Several authors have stressed the importance of explainability in medical AI for a broad spectrum of stakeholders, including AI developers, physicians and other healthcare providers, patients, health insurance, administrators, lawyers, and policymakers. While the need for explanation certainly varies between individuals and groups, especially for clinicians, explainability has been considered crucial [5,25,26,28,48]; see Section 3.2.

Explainability helps developers and clinicians check whether the technology does what it is supposed to do, i.e., relies on the relevant factors for its output. Explanations may help avoid errors and bias, improving clinical decision making and avoiding patient harm resulting from misclassifications. In contrast, with opaque AI-based tools, clinicians do not know what the output is based on and cannot adequately communicate medical decisions involving these tools to patients (see Section 3.2). Furthermore, it does not seem fair to make medical professionals morally accountable or legally liable in case of harm that results from decisions based on black box AI output [20,25,26].

According to Tianhua Chen and colleagues, the reasons for the central relevance of explainability in medical AI are obvious and powerful [31] (p. 3): “no medic or healthcare provider, who would ultimately be responsible for their actions towards patients, could take at face value the outcome of a computer-based system, no matter how much knowledge, expertise or intelligence is encoded in it”.

In clinical contexts, it is not just clinicians but also patients for whom explainability has been considered of central importance. Patients need an adequate amount of information and understanding to be able to give their informed consent and trust their doctors. All of this is difficult to achieve with black box AI tools, however (see Section 3.7).

In each of the use cases introduced above, different stakeholder groups need to be considered, and the role of explainability for the stakeholder groups varies.

In the emergency call use case, the medical dispatchers play a crucial role, as they are the users directly interacting with the tool. It is plausible to assume that lack of explainability and lack of trust may have negatively influenced, if not prevented, the tool’s usage. Against this background, the hypothesis that adding explainability could increase dispatcher compliance with the tool’s recommendations seems worth pursuing (see [28]). In view of the prevalence of time constraints in 112 emergency calls, any form of explanation would have to be provided instantly, and dispatchers would have to be able to see easily and directly whether the explanation provided is plausible.

In the second use case, explainability plays a central role for medical doctors and patients. Medical doctors need explanations to gauge the plausibility of the prediction and to communicate in a meaningful way with their patients about the AD diagnosis. Patients can be expected to request convincing and plausible explanations, given the severity and far-reaching implications of an AD diagnosis.

In the example of AI-supported diagnosis of adult ADHD, explanations are directed toward junior and senior clinicians. The knowledge-based explanations help junior clinicians to distinguish patients with complex cases from those with straightforward cases and to communicate this distinction to them. For senior psychiatrists, explanations may be useful in that they hint at why the patients were referred to them.

A broad spectrum of potential future addressees of AI-provided explanations has been suggested in the use case that automates moral decision making. These include patients, clinicians, ethics committee members, and students in educational settings. In all of these contexts, explanations would certainly be needed to make the tool output plausible and convey it to patients.

### 3.2. Explainability, Medical Decision Making, and Autonomy

There has been an ongoing debate on the various ways in which the use of AI-based CDSSs may influence medical decision making. On the one hand, the technology has the potential to significantly improve medical decision making in that its complex data processing brings in a multitude of factors that may otherwise not be considered. However, the involvement of AI in medical decision making also comes with downsides. In particular, AI-based CDSSs have been characterized as an additional component, a “third authority”, that is difficult for medical professionals and patients to ignore and thus may negatively influence clinical decision making, doctor–patient relationships, and informed consent [12,16,17,48,49,78,79,80]; see also Section 3.7.

In addition, there is a more general question of how the use of AI tools may influence decision-making standards in medicine and healthcare. If AI-based decision making proves to be more accurate, more reliable, more efficient, or more cost-effective than traditional decision making, this may shift standards, and medical professionals may end up having an obligation to integrate AI in their decision making or to rely on AI [13,17,20,49].

Knowledge of the relevant factors of a clinical decision-making situation is fundamental for doctor and patient autonomy, informed consent, moral accountability, and legal liability. Compared with non-explainable AI, explainability makes the AI output intelligible to human users. Several authors have stressed that it is explainability that allows medical professionals, patients, and other users to assess the model output and utilize it as a “second opinion” that supports clinical decision making. Accordingly, explainability is needed for AI-based CDSSs to be decision *support* systems and not tools that substitute for human decision making [16,25,26,28,41]. The point was made that non-explainable AI threatens the autonomy of clinicians because it is difficult for them not to follow the non-negotiable AI output. This may promote a tendency to decide in line with the tool to avoid potential litigation (“defensive medicine”) [16,49].

Furthermore, several authors have argued that clinicians and patients need AI explainability to trust the models and that lack of trust may slow down or limit the adoption of AI in medicine [25,26,81,82]. Accordingly, explainability in AI may facilitate the use of AI-driven technology in medical contexts.

In the first use case that involves a ML tool to identify cases of cardiac arrest in emergency calls, lack of explainability seems to have played an essential role in the dispatchers’ lack of compliance. With the black box tool, the dispatchers could not make autonomous decisions regarding individual calls. There was no way for the dispatchers to consider the classifications provided by the tool constructively, they could only decide whether they wanted to rely on the system.

The other three use cases give different examples of tools that provide some form of explanation. While empirical studies are lacking, it is plausible to assume that the explanations have the potential to support clinicians in decision making.

The AI-based tool in the second example is designed with the aim of improving the identification of the early stages of AD. Explainability is a central component of any AI technology involved in a sensitive context like AD diagnosis. It would be inadequate to convey a diagnosis of early AD to a patient without giving and explaining the relevant medical details. The post hoc explanations provided by LIME indicate the brain regions relevant for the AD predictions. The medical relevance of these explanations is unclear, however. While LIME identifies a number of regions as relevant for the AD predictions, these regions differ considerably between the four different CNN models tested. In this use case, it is central for clinicians to bring in their medical knowledge to evaluate the reliability of the tool output (see Section 3.3).

In the ADHD use case, the purpose of the tool is to help separate the clearcut cases of adult ADHD from the more complex ones. Clinicians can quality check the medical knowledge-based explanations and use them to explain to patients why and how they rely on the tool output (positive, negative, inconclusive). It is the introduction of the knowledge model that enables the hybrid system to not only make yes or no predictions, as would be performed by a ML model alone, but to bring in a third option. The third option, which indicates an inconclusive result, is of crucial clinical relevance. Instead of making an error-prone yes-or-no classification, the tool is less error prone when it identifies complex cases and recommends consultation by a senior psychiatrist. Insofar, the hybrid model has the potential to function as a decision *support* tool for medical professionals with which they can interact. It does not consider the full spectrum of patient-related information, however. For fully autonomous decision making concerning patients, clinicians would need additional information that normally results from direct interaction with patients, such as information related to body language or lifestyle.

The ethics committee use case seeks to automate moral decision making in the context of complex medical cases. Based on previous ethics committee decisions, the model provides treatment suggestions. Explainability is of crucial relevance in this use case. It allows users—clinicians, patients, ethics committees—to check whether the model output aligns with their central values. Without explainability, a classification like “morally right” or “morally wrong” would be utterly unacceptable (see Section 3.6). However, the very approach of automating moral decision making entails giving up autonomy about value judgments. Autonomy in the context of moral decision making implies that individuals make their own decisions based on their preferences and values, instead of receiving AI-derived recommendations.

### 3.3. Types of Explanation

In many contexts, the sentence “the machine calculated it” is a perfect explanation. With us knowing the rules of multiplication, we consider 8 × 8 = 64 an absolutely satisfying explanation. We would expect that a machine output of 32,984 × 5398 = will be correct and perfectly well explained by the rules of multiplication. The situation is different with complex machine learning approaches such as neural networks. Here, even developers and experts are unable to understand how the system comes up with its output as much more complex calculations are involved. Furthermore, our human explanations are not necessarily about probabilities and numbers but about concepts and meanings; they build on knowledge and experience. It can be a challenge to bring these in line with numbers and probabilities.

Integrating explainability into complex AI technology has been seen as a way to reduce or limit these issues, especially in sensitive fields like medicine. However, from an empirical perspective, the relevance of explainable AI in medicine is unclear, as is the question of what counts as an adequate explanation [28,83].

Several different types of explanations can be discerned. This section will introduce the types of explanations that matter in the context of the use cases.

#### 3.3.1. Medical Knowledge

It seems plausible to assume that explanations involving medical knowledge have the potential to be perceived as valuable because they are closely related to the way medical professionals and patients think.

The ADHD use case directly involves medical knowledge in that the model indicates the patient questionnaire and interview assessments and scores it relies on. However, not all types of medical knowledge valuable for diagnosing adult ADHD are considered. The automated process lacks information commonly resulting from direct doctor–patient communication, such as patient posture, facial expression, or lifestyle-related aspects. In addition, for the knowledge model, interviews with only one expert were taken into consideration. Relying on a larger number of experts would be advisable to avoid idiosyncratic rules, shortcuts, or biases.

In the use case involving automation of moral decision making, medical knowledge and knowledge concerning ethical principles (beneficence, nonmaleficence, autonomy), as well as individual values and preferences, are central. However, the concepts the tool relies on are poorly defined. For example, when an indication is given that autonomy or patient best interest played a central for a tool recommendation, it lacks specification on what is meant by “autonomy” or “patient best interest” in the context of the medical case. Users with different sociocultural backgrounds may have different understandings of the central concepts involved (see Section 3.6). Furthermore, it seems arbitrary to consider a model output of 0.5 or above as a recommendation supporting the intervention in question.

#### 3.3.2. Feature Importance

Feature importance refers to a certain factor’s relevance for a model. Features with higher scores have a larger impact on the model output than features with lower scores. Information on feature importance helps users check whether the model output is based on what it is expected to be based on [81,83].

In the AD diagnosis example, LIME provides a heatmap that directly indicates feature importance, i.e., the relevance of the various brain regions for AD prediction. Given that LIME flags brain regions based on correlations and does not indicate any causal role, some brain regions flagged by LIME may not be relevant for AD (see the section on correlation below).

In the ADHD case, no information on feature importance is given concerning the relevance of the different scores for ADHD prediction in individual patients. Regarding the general way the model arrives at its output, however, the if-then rules indicate feature importance in that the scores for certain surveys or interviews are more relevant than the scores for others. For example, the hierarchy of rules is so that low DIVA scores for ADHD are of the highest relevance for the model and imply the prediction “no ADHD”.

In the last example involving moral decision making, the model gives an indication of the causal relevance of the various factors for the recommendation in question. For example, in a specific case analysis ([71], p. 14), it indicated that the activation for “follow patient’s best interest” was almost 1, whereas the activation for autonomy was approximately 0.2 as compared to approximately 0.1 for beneficence. While this gives an idea of the relative importance, it is not clear how to bring the activation pattern in line with the way the various factors would be considered in human moral decision making.

#### 3.3.3. Correlation (Post Hoc Explanation)

Post hoc explanations provided by methods like LIME or SHAP are about correlations. They indicate the relevance of certain features for the model output, but not whether the identified features play a causal role in the prediction.

Adding post hoc explainability can be seen as having important advantages over black-box models, as revealed by famous cases like the one involving huskies and wolves where irrelevant factors proved central for the classifications [65]. However, adding second models like LIME or SHAP also has several downsides. The second model does not necessarily represent the working of the opaque first model correctly, so that the explanations provided may be incorrect, misleading and time-consuming [28,32,48].

In addition, post hoc explanation approaches come with what has been called an “interpretability gap” [32,48,51]. While post hoc explanation techniques like LIME or SHAP indicate the relative importance of certain features for the prediction, they do not provide an explanation for why these are marked as important. Clinicians must fill the gap and provide an interpretation based on their clinical knowledge. In doing so, there may be a tendency for clinicians to inadequately assume that the model considers the same features relevant as they do (confirmation bias). Insofar, the explanations may seem much more reassuring than they actually are [51].

Among the three examples that involve explainable AI, only the AD use case relies on post hoc explanations. The explanations in the form of heat maps indicate the relevance of specific brain regions for the model output. The heat maps indicate correlation, not causation. For the use case, this implies that it is not necessarily the case that the flagged brain regions are medically relevant for the early stages of AD. This is further underlined by the fact that the LIME-provided heatmaps differ considerably depending on the opaque CNN model on top of which LIME is run. The reasons for these differences are not known, and neither is it known whether these differences trace back to the CNN models or to LIME. It is up to the clinicians to assess the LIME output based on their medical knowledge and clinical experience.

#### 3.3.4. Causation

Given the problems described above with correlation-based explanations, causal explanations have been considered preferable by a broad spectrum of authors, especially in high-stakes scenarios such as the medical domain [32,84]. This ensures that the explanations provided by a model indicate the correct reason behind a prediction. In contrast, others have argued that causal explanations are not always needed, given that medical explanations are not necessarily causal explanations [25,50,85].

Causal explanations are given in the ADHD use case and the moral decision-making use case. In the ADHD use case, a causal explanation is provided: the hybrid model indicates the surveys and scores the output is based on. However, it does not consider other relevant factors, such as body language or appearance. Also, the medical knowledge involved in the ADHD case is not causal knowledge in the strict sense. It is generally accepted medical knowledge that usually is adequate. It does not say anything about why someone has ADHD.

The moral decision-making use case involves a causal network that indicates the causal relevance of the various factors for its output. In contexts that involve moral decision making, causal explanations are important for users to understand whether the tool output aligns with their fundamental values and assumptions. The explanations given about the role of certain concepts for the model output are based on how these concepts are shaped and thus rely heavily on quantifiable factors, however. The way clinicians or patients understand complex concepts like beneficence, non-maleficence, or autonomy may deviate considerably from the way these concepts are implemented in the database and framed by the tool.

### 3.4. Accuracy Versus Explainability

Complex ML models, such as deep neural networks, often have higher performance and accuracy than more traditional approaches with built-in explainability, such as linear regression or logistic regression. Concerning the choice of the model, in many contexts, this involves a tradeoff between explainability and accuracy and raises the question of the relevance of explainability as compared to accuracy [23,32,86].

While several authors have stressed the relevance of explainability for medical AI [9,26,32,49], others have questioned its importance and instead emphasized the need for quality standards for clinical validation involving accuracy and reliability [25,50,51,85]. They argued that the more accurate and reliable a tool, the less there will be need for explainability. Accordingly, explainability may not be needed for high validity tools. This position has been called the “Validation View”, as opposed to the “Explanation View” [48]. Others have underlined that there is not necessarily a conflict between accuracy and inherent explainability, however [32,86].

With regard to balancing the demands for accuracy and explainability, there are clinical applications and contexts where explainability is more relevant as compared to accuracy than in others. It is plausible to expect high explainability needs in medical professionals and patients during the development and introductory phases of complex new AI-based tools. Once a tool’s accuracy and reliability have been established and undergo regular monitoring, there may be less need for explanations, especially in the case of low-risk applications. Also, it is imaginable that explainability may be considered less relevant the longer certain tools have been in use. Routine use may come with less explainability expectations. These are questions that need empirical investigation.

When it comes to specific applications and use cases, it is the choice of the model that shapes the accuracy and explainability of the approach. In this choice, adequate consideration of the tradeoff between explainability and accuracy certainly is central.

In the AD use case involving black box CNNs and post hoc explanations, it is unclear how adding LIME affects accuracy. It seems plausible to assume that the LIME model adds inaccuracy risks, as the LIME-provided explanations are not necessarily accurate. Omitting the LIME model would allow for a direct comparison of the performance of the approach with and without post hoc explanations.

In the other two use cases with built-in explanations, it remains to be seen whether black box approaches with similar functions could be developed and what their performance would be. In these two use cases, the relevant reference point is the accuracy of the current practice that does not involve the use of AI, and not the performance of a hypothetical black box tool, however.

In the ADHD use case, the hybrid tool that combines a ML model and a knowledge-based model has higher accuracy than the ML model alone, i.e., the addition of an explainability component increases the overall accuracy. It is unclear, however, whether the tool is more accurate in executing the task than the clinicians, i.e., whether the addition of the hybrid tool improves or diminishes the accuracy of the overall process.

In the moral decision making use case that provides built-in causal explanations, there is no conflict between accuracy and explainability in the strict sense. However, the use case involves the fundamental problem that there is no objective accuracy when it comes to moral decision making. Insofar, it is an unresolved question what high accuracy could mean with regard to moral decision making in medical contexts (see Section 3.6).

### 3.5. Epistemic Authority and Automation Bias

AI technology has been perceived as an epistemic authority in the sense that given the broad database and training, there is reason to believe that the AI output is correct [87]. In line with this, several authors stressed that medical AI technology, especially black box AI, will be considered as coming with epistemic authority and that human users may feel compelled to follow the AI output [16,49,78]. Accordingly, the perceived epistemic authority of AI technology favors automation bias in healthcare, which can be described as a tendency to over-rely on automation and to follow suggestions from automated tools instead of critically reflecting on their plausibility. Automation bias when using AI-based CDSSs may tempt clinicians to disregard contrary indications and data and reverse a correct decision they have already made and thus introduce errors [88,89,90].

Particularly concerning black box CDSSs, considerable concern has been raised that human decision makers may rely too heavily on the technology, especially in situations of time pressure or heavy workload. This overreliance may lead to misdiagnoses and other medical errors and comes with deskilling risks and negative implications on clinician performance, decision-making capabilities, and deterioration of AI performance [16,17,78,79,80,89,90]. In this context, the term “AI paternalism” was coined [12,17,49,91,92]. The term has been used to refer to AI technology making independent decisions without the involvement of patients [91,92], or even as a form of epistemic paternalism that applies both to clinicians and patients [49] (footnote 26 on p. 366).

Given that the black box nature of AI models and training processes has been considered to be among the main contributors to automation bias, adding explainability is expected to reduce automation bias. With the explanations provided, clinicians, patients, and other users may be empowered to balance what weight they want to give to the predictions and recommendations. Accordingly, explainable AI allows users to ponder the AI output and critically reflect on it (see Section 3.2 and Section 3.7).

On the other hand, however, there is a risk that the tendency to overly rely on the output of AI tools can even be furthered by tools that provide explanations. The fact that explanations are given may make the tool seem more convincing and confer additional authority to its predictions and recommendations. The explanations may seem much more plausible than they actually are. These concerns align with a recent clinical vignette-based study on the effects of AI involvement on clinician diagnostic accuracy [89,93]. The results showed that providing image-based explanations only to a very limited extent helped alleviate the negative impact of systematically biased models. According to the study’s authors, one reason for this only minor effect of adding explainability can be seen in the limited capabilities of clinicians to detect bias [89,93].

Aspects related to epistemic authority and automation bias play out differently in the contexts of the use cases. While in each of the four use cases, the tool was developed to support human decision making and not to substitute for it, in each of the use cases, the risk that users rely too heavily on the tool plays a role.

According to the theoretical discussion outlined above, the emergency call use case is heavily prone towards automation bias. Given the black box nature of the tool and the time pressure in emergency calls, it might have been expected that dispatchers have a high tendency to rely on the tool. The opposite was the case. A plausible explanation for the lack of dispatcher compliance could be that the dispatchers considered the tool’s epistemic authority to be questionable, either because of the lack of AI-provided explanations, the lack of proven clinical validation, or general mistrust towards AI. Also, in contrast to the other use cases, it was relatively easy to ignore the tool, as the alarm would go off rarely, i.e., at a very low percentage of emergency calls.

Notably, empirical data on how users interact with the AI are absent in the three other use cases. While the explanations may be expected to be helpful, they may bear the risk of seeming overly convincing and obfuscating the possibility of misclassifications.

This is true for the AD use case, in which no empirical data exist on how clinicians interact with the tool and what level of epistemic authority they attribute to the heatmap-based explanations that indicate the relevance of the various brain regions for AD prediction. Given the nature of post hoc explanations, it will be crucial for clinicians to have the capability to critically assess the tool output.

In the ADHD use case, the tool groups the patients into straightforward cases to be seen by junior clinicians and complex cases for senior clinicians. This distinction is not made by the junior clinicians, but by the tool. In this setting, the level of epistemic authority attributed to the tool is located right between the epistemic authority of a junior clinician and a senior clinician. It remains to be seen whether there is a risk for junior clinicians to over rely on the tool outcome.

Issues around epistemic authority and automation bias are particularly striking in the last use case. In situations involving complex moral decision making, where doctors, patients, or ethics committees feel uncertain about how to decide, an AI-based CDSS may be welcomed as providing helpful advice. There is a clear risk that patients, doctors, or ethics committee members assume that the tool comes with higher epistemic authority than they themselves, as it represents a large body of previous ethics committee decisions. However, the tool’s epistemic authority is highly questionable because there usually is no “objectively” correct answer in complex moral decision-making contexts (see Section 3.6).

### 3.6. Individual Values and Preferences

Healthcare-related decisions are usually not only about facts. Individual preferences and values, as well as the sociocultural context, lifestyle, and life situation of the individual patients, also play a role, sometimes to a more considerable extent, sometimes to a smaller extent. For example, a treatment that comes with the risk of side-effects that involve swelling of the fingers or limbs will almost certainly be considered differently by a pianist, a professional runner, or a teacher. Adequately considering individual values, preferences, and sociocultural contexts in AI-based CDSSs and indicating the role these aspects have for predictions and recommendations are fundamental challenges [12,49,94].

In some of the use cases, values and individual preferences do not seem to have a high priority, but they do play a role in each use case. In emergency situations like emergency calls in the first use case, there is a general consensus that taking immediate life saving measures is of utmost importance. It can be expected that compliance with the ML tool output would increase the rate of cardiopulmonary resuscitations being administered, given the higher sensitivity of the tool as compared to the dispatchers. While this is intended to save lives, there may also be conflicts with patient advance directives.

In the AD use case, values come in concerning the standards for clinical validation, the quality of the database, the predictive accuracy, and doctor–patient communication. The value-related aspects of communication with patients include how the AD diagnosis and the anticipated mental decline are conveyed to patients, and how the role of the model, the model accuracy, and the possibility of false positive and false negative results are disclosed to patients.

With the involvement of the ADHD tool to support the diagnosis of adult ADHD, the triage step of the process focuses on standardized tools, whereas direct doctor–patient interaction and communication are limited. It may be questioned whether patient preferences, values, and sociocultural contexts can adequately be considered in the seemingly easy, clearcut cases where there is no senior psychiatrist involved. An empirical study will be needed to find this out.

The role of values and individual preferences is most apparent in the last use case that involves the automation of moral decision making. Here, individual patient values and preferences serve as direct input for the CDSS. In the user interface, in categories like beneficence, non-maleficence, and autonomy, users can fill in their data and preferences regarding quality of life, life expectancy, willingness to take risks, priorities of life expectancy over quality of life, and other aspects. Insofar, there is a way for the model to incorporate at least some patient values and preferences. However, only a small part of a person’s values and preferences can be considered. In addition, while the approach focuses on quantifiable aspects like priority of life expectancy versus quality of life, it tends to dismiss qualitative aspects, hopes, emotions, worries, and fears. Adequate consideration of individual values and preferences is further complicated by the fact that different individuals with different sociocultural backgrounds may have different understandings of the central concepts involved.

Explainability certainly is crucial to the approach in the last use case, because users will want to know whether the tool’s recommendations are in line with their own preferences and values. It is questionable, however, whether individual human moral decision making can ever be automated in a meaningful way [73,74,75,76,77]. This is particularly true as in most cases there is no clear and unambiguous morally right or morally wrong decision or position. Moral decision making strongly depends on the decision maker’s views, values, preferences, and life situation. The assumption that an AI can come up with objectively correct classifications, predictions or recommendations in these contexts is very problematic.

In addition, there is a clear risk that a tool like this ends up streamlining decisions that would otherwise be made in a more diversified way [76]. This risk exists in several regards: (a) The tool relies entirely on a Western approach to medical ethics. In line with the Western liberal tradition, the role of informed consent and advance directives is stressed. In contrast, factors like individual life goals or the role of family members lack consideration. (b) Furthermore, its use by ethics committees and clinicians, or in medical education, implies an unwarranted “standardization” of medical decision making and medical ethics, in the sense that a tool that relies on past ethics committee decisions is supposed to provide generally accepted recommendations. (c) Also, there is a danger of perpetuating current or past positions on medical ethics and decision making in complex medical cases into the future.

### 3.7. Doctor–Patient Relationships and Patient Autonomy

The clinical use of AI-based CDSSs can be expected to modify doctor–patient relationships in various ways. As several authors have stressed, in doctor–patient interactions, the AI tool may be perceived as a “third authority” that is difficult to ignore [12,16,17,48,49,78,80]. Accordingly, this expansion of dyadic doctor–patient relationships to triadic doctor–AI–patient relationships entails modifications in the roles of medical doctors and patients, as well as their autonomy and epistemic authority. The higher the relevance attributed to seemingly objective data, the less importance and space is expected to be given to clinical experience, intuitions, patient testimonials, patient experience, and doctor–patient communication.

Given the potential implications of CDSSs on doctor–patient relationships, respect for patient autonomy requires disclosing the involvement of AI-based CDSSs and providing adequate opt-out options, wherever possible.

For patients, the role of AI-based tools and the AI output may be difficult to understand if not seem entirely unintelligible. This may impede patient involvement in medical decision making and patient autonomy [12,49,80]. McDougall [12] and Bjerring & Busch [49] argue that relying on black box AI tools for medical decision making is in conflict with patient centered care and shared decision-making standards. Accordingly, it reduces meaningful patient involvement and limits patient autonomy and adequate consideration of patient values, all of this makes meaningful patient involvement and shared decision making difficult if not impossible [12].

Against this background, explainability in AI may reduce the risk of AI to influence doctor–patient relationships, autonomy, and informed consent negatively. While a risk exists with both explainable and black box systems, it is easier to integrate explainable AI in medical decision-making processes and informed consent procedures. With explainable AI, clinicians have something they can tell their patients, who can gain some understanding of what the model output was about and why their doctors consider it important, relevant, or correct. Explainability in medical AI seeks to support doctor–patient communication, meaningful patient involvement, and shared decision making. It enables clinicians and patients to collaboratively reflect on the AI output and effectively bring in the patient perspectives and preferences in the decision-making process. Accordingly, an explainability-based approach facilitates patient autonomy and trust [12,18,25,26,49].

Several authors have stressed that explainability is required for clinicians and patients to trust the tools. The point was made that lack of trust may slow down or limit adoption of AI in medicine, whereas explainability may help increase acceptability among medical professionals and patients [25,26,81,82].

However, meaningful consideration of the role of AI technology in doctor–patient-relationships requires adequate understanding of the technology, its benefits and limitations, not only by clinicians but also by patients [17,25,26,49]. Patients are not technology experts. If they are to make autonomous decisions around medical recommendations involving AI tools, they must be provided with a considerable amount of information. For medical professionals, understanding the involvement of AI technology and translating it into information that patients can understand is a crucial new task and a challenge.

In contrast to this widespread view, other authors argued that patients may not need a lot of information about AI tools, and that it may be enough for them to know that normally, the tool is reliable [25,50,85]. According to this perspective, the relevance of explainability in AI-based CDSSs for patients and doctor–patient-relationships has been overstated.

It is important to stress that in none of the use cases has there been an analysis of the implications of the use of the AI tool on patients and doctor–patient relationships. While this is certainly due to the fact that the tools presented in the use cases are all in the relatively early stages of their development, empirical research on this question will undoubtedly be needed.

In all cases, patients would have to be informed about the involvement of an AI-based CDSS and would have to be given the option to opt out of using the tool. While this is not possible in the immediate context of an emergency in the first use case, these options are available in the other three use cases.

The emergency call use case does not involve doctor–patient relationships in the strict sense.

In the AD use case, the implications of a potential clinical deployment of the AI-based tool on doctor–patient relationships are unknown. The LIME-based explanations seek to enable clinicians to convey to patients some indication of why they receive an AD diagnosis. It will be important for clinicians and patients to understand the downsides of these correlational explanations and to clearly communicate existing uncertainties. It remains to be seen whether clinicians may be perceived as less of an authority if they depend on this type of technology or whether AI involvement may increase a doctor’s reputation in the case of highly accurate predictions.

In the ADHD use case, with a clinical deployment of the hybrid tool that separates patients in straightforward and complex cases, a considerable percentage of patients would no longer see a senior clinician. For this group of patients, standardized assessments would become more important with less direct doctor–patient interaction, rendering factors like clinical experience and doctor–patient communication less relevant than they would otherwise be for diagnosing ADHD. The knowledge-based explanations provided by the tool may confer the impression that all relevant factors have been covered, but the model may miss aspects a senior expert would have identified. Empirical studies are needed to determine whether patients would appreciate the tool’s benefits and the shortening of waiting times or criticize the new setting as coming with a decrease in the quality of consultations and doctor–patient relationships.

In the moral decision-making use case, if the tool were to be used together with patients, extensive doctor–patient interaction would be needed to find out about the information to feed into the ML model. Collecting all the information to adequately represent the patient perspective would probably require very detailed and empathetic doctor–patient conversations. With this type of doctor–patient-communication in place, there is no need for AI-based moral decision making, though. If the tool were used without direct patient involvement, it would end up substituting for doctor–patient communication. Particularly in complex situations involving moral decision making, meaningful doctor–patient interaction is necessary, however. Outsourcing the decision making to a ML tool seems counterproductive. Another suggested possible form of utilization is for patients and relatives to directly interact with the tool, again substituting for doctor–patient communication.

Even though, in this use case, explainability allows us to communicate how the treatment recommendations came about, it seems extremely difficult for users to understand the limitations of the tool and the role it might have in their own decision making. This is particularly problematic if patients directly interact with the tool. In all these potential use contexts, negative implications on patient autonomy have to be expected (see Section 3.5 and Section 3.6).

## 4. Limitations

This research has several limitations. It is a literature-based study that relies on published literature to analyze the role, relevance, and ethical implications of explainability in AI-based CDSSs by discussing four use cases. The author is not part of the research groups that develop the AI-based tools in the four use cases. Also, the use cases involve tools that are currently under development. Detailed studies on how medical professionals and patients interact with the tools in clinical contexts do not exist yet. This implies that in this literature research-based study, to discuss the ethical and user-related aspects of explainability in the use cases, it has been necessary in part to infer from and build on related research. As the author of this article is not directly involved in any of these use cases, it was not possible for the author to conduct an interview study or collect data on the perspectives of medical professionals and patients, how they interact with the tools, or how the tools influence decision making and doctor–patient relationships. In addition, recent modifications and improvements of the technology may not have been covered in this article.

## 5. Conclusions

As can be seen from the above discussion, explainability (or the absence of explainability) plays a vital role in the four examples of AI-based CDSSs. In each of the three use cases that seek to provide meaningful explanations, it is plausible to assume that explainability has the potential to facilitate the integration of the technology in medical decision-making processes and to reduce potential adverse effects of automation on doctor–patient relationships, autonomy, and informed consent. The explanations may help medical professionals to critically reflect on the model output and constructively consider it in their clinical reflection. The use cases also revealed various ways in which there is a risk of overreliance on AI-based tools. It remains to be seen whether the type and quality of the explanations are sufficient for users to rely on the systems or whether they need different types of explanations.

In addition, explainability and the various aspects related to explainability play out differently in the different use cases. A careful case-by-case analysis is needed on what type and amount of information is important in the respective context, as the answer to what is a good and useful explanation clearly depends on the tool and its use context. While explainability may be of crucial importance in certain AI-based CDSSs and use contexts, it may be less relevant or even dispensable in others. A case-based analysis will allow us to identify the role and relevance of explainability of AI-based tools in their respective contexts and to reflect on how explainability can be implemented.

Importantly, the case studies discussed above are all experimental approaches. Currently, there is only limited knowledge on the tools, their potential future development, and their implications. This lack of empirical data on the role, relevance, and implications of explainability in medical AI holds not only for the four use cases but is an issue more broadly speaking. Not much is known about what type and amount of explanation is needed in AI-based CDSSs, how explainability can be adequately implemented, and how explainability in AI-based tools would influence clinical practice. While it is plausible to assume that explainability will have positive effects in many use contexts, it will be crucial to avoid incorrect and misleading explanations as well as unnecessary information overload that may distract from relevant factors.

Given this, studies are clearly needed to investigate the role and relevance of explainability in specific medical AI applications. This involves both the technical and user perspectives and includes questions like: Who needs explainability? What type of explainability is needed or considered adequate? How does explainability influence medical decision making and doctor–patient relationships?

In the future, this research will be expanded to include additional use cases to obtain a more comprehensive picture of the role and relevance of explainability in AI-based CDSSs. In addition, given the current lack of empirical studies on how medical professionals and patients interact with explainable medical AI, future research seeks direct collaboration with technical and medical research teams to integrate ethical aspects and user experience-related questions early in the research design process.

## Figures and Tables

**Table 1:** Overview of the role of explainability in the use cases. (-: no relevance; x: relevance; xx: strong relevance).

Use Case	Emergency Calls	Alzheimer’s Disease	Adult ADHD	Moral Decision Making
Type of explanation	No explanations	Post hoc explanations	Knowledge-based explanations	Explanations involving complex concepts
Immediate users	Medical dispatchers	Medical doctors	Junior psychiatrists	Ethics committees, patients, clinicians, medical students
Support for human decision making	-	x	xx	x
Relevance of tradeoff between explainability and accuracy	-	x	-	-
Risk for automation bias	xx	x	x	xx
Role of patient values	-	-	unclear	xx
Relevance for doctor–patient communication	-	unclear	x	xx

## Data Availability

Data are contained within the article.
